# Human Adipose-Derived Mesenchymal Stem Cells in Cell Therapy: Safety and Feasibility in Different "Hospital Exemption" Clinical Applications

**DOI:** 10.1371/journal.pone.0139566

**Published:** 2015-10-20

**Authors:** Sophie Vériter, Wivine André, Najima Aouassar, Hélène Antoine Poirel, Aurore Lafosse, Pierre-Louis Docquier, Denis Dufrane

**Affiliations:** 1 Endocrine Cell Therapy, Centre of Tissular and Cellular Therapy, Cliniques Universitaires Saint Luc, Brussels, Belgium; 2 Plastic Surgery, Cliniques Universitaires Saint Luc, Brussels, Belgium; 3 Orthopedic Surgery, Cliniques Universitaires Saint Luc, Brussels, Belgium; 4 Center for Human Genetics, Cliniques Universitaires Saint Luc, Université catholique de Louvain, Brussels, Belgium; Second University of Naples, ITALY

## Abstract

Based on immunomodulatory, osteogenic, and pro-angiogenic properties of adipose-derived stem cells (ASCs), this study aims to assess the safety and efficacy of ASC-derived cell therapies for clinical indications. Two autologous ASC-derived products were proposed to 17 patients who had not experienced any success with conventional therapies: (1) a scaffold-free osteogenic three-dimensional graft for the treatment of bone non-union and (2) a biological dressing for dermal reconstruction of non-healing chronic wounds. Safety was studied using the quality control of the final product (genetic stability, microbiological/mycoplasma/endotoxin contamination) and the *in vivo* evaluation of adverse events after transplantation. Feasibility was assessed by the ability to reproducibly obtain the final ASC-based product with specific characteristics, the time necessary for graft manufacturing, the capacity to produce enough material to treat the lesion, the surgical handling of the graft, and the ability to manufacture the graft in line with hospital exemption regulations. For 16 patients (one patient did not undergo grafting because of spontaneous bone healing), in-process controls found no microbiological/mycoplasma/endotoxin contamination, no obvious deleterious genomic anomalies, and optimal ASC purity. Each type of graft was reproducibly obtained without significant delay for implantation and surgical handling was always according to the surgical procedure and the implantation site. No serious adverse events were noted for up to 54 months. We demonstrated that autologous ASC transplantation can be considered a safe and feasible therapy tool for extreme clinical indications of ASC properties and physiopathology of disease.

## Introduction

Cell therapy has recently gained more interest from scientists and clinicians. It offers new therapeutic tools and hope for patients who have not experienced success with classical treatments by proposing "personalized" therapies for selected indications. In some cases (for example, radiotherapy, diabetes, burns, etc.), fibrotic tissue is observed with a lack of tissular vascularization and irrigation, poor stromal cell recruitment, and a paucity of growth factors. In these cases, cell therapy is proposed to restore the physiology of the injured tissue by the induction of tissue vascularization [1], immunomodulatory effects [2,3], and secretion of growth factors promoting tissue remodeling and regeneration [4–7].

Among the different sources for cell therapy, mesenchymal stem cells (MSCs) are the most studied because they offer the advantage of being isolated from adult donors and demonstrate the capacity to differentiate into multiple tissues, including bone, fat, skeletal muscle, and cartilage [8,9]. MSCs were first isolated from bone marrow by Friedenstein more than 50 years ago [10]. More recently, a new source of MSCs was proposed, the adipose tissue [11]. Adipose mesenchymal stem cells (ASCs) are easily accessible in abundant quantities and can be collected by a minimally invasive procedure. Adipose tissue-derived stem cells showed properties similar to those of bone marrow-derived MSCs and showed even better isolation reproducibility and higher proliferation capacity [12,13]. Moreover, ASCs demonstrated four properties that could be helpful in cell therapy: angiogenicity [1,13–16], osteogenicity [13,17], immunomodulation [18], and promotion of tissue remodeling [4,5,16].

First, to initiate tissue remodeling, the product needs to be vascularized for appropriate oxygen and nutrients to be supplied from the blood stream. In this context, mesenchymal stem cells, and mainly ASCs, are of particular interest because they are able to secrete growth factors promoting angiogenesis (VEGF, HGF, PDGF, FGFb) [7,15,16,19,20]. Interestingly, these pro-angiogenic properties are maintained after osteodifferentiation [13]. Therefore, the use of ASCs is highly justified to induce the revitalization of the tissue, both in bone and skin reconstruction. Moreover, ASCs were also shown to secrete bone morphogenic protein 2, which is involved in bone remodeling and bone formation [21]. In addition, ASCs were shown to secrete fibroblast growth factor-2, a pro-angiogenic factor involved in the wound healing process [22,23], keratinocyte growth factor, which is a growth factor with paracrine effects on cells implied in wound healing [24,25] and insulin-like growth factor 1, which is important in wound healing because it promotes wound re-epithelialization and granulation tissue formation [26–29]. Therefore, in this study, ASCs were seeded on a collagen membrane to promote wound healing by the induction of tissue vascularization and tissue remodeling.

Cases of hospital exemption treated in this study all had physiopathology of disease characterized by a lack of spontaneous tissue remodeling, principally attributable to a lack of growth factors required to initiate tissue vascularization, stem cell recruitment, proliferation, and differentiation and to control inflammation. In this context, the properties of ASCs were perfectly adequate for the physiological effects of the graft and the needs of the patient.

In response to a specific request from surgeons confronted by patients with "untreatable" pathologies and failure of conventional treatments, we tried to take advantage of these four properties of ASCs to develop new therapeutic approaches. Two types of cellular therapy products were required for bone non-union and non-healing chronic wounds. There was a need for the development of a malleable three-dimensional (3D) cell therapy product with osteogenic and angiogenic properties for bone reconstruction in orthopedic surgery [13,17] and a suturable biological dressing for the promotion of angiogenesis in the wound bed and dermal regeneration [16].

These products were developed to treat specific patients with end-stage pathologies in the context of hospital exemption. The safety and feasibility remain the first questions to be studied.

The term "safety" implies minimizing the risk/benefit ratio, which takes into account the improvement of the quality of life expected for treated patients and the risks associated with the procedure. These risks include the following: microbiological safety of the product; genetic stability of the product; absence of adverse events after cell procurement and implantation; homing of transplanted cells to the site of injury; absence of ectopic tissue formation; *in vivo* oncogenic/tumorogenic safety of the cellular product; and control of host immune response against the transplanted cells. Although risk reduction can be achieved by the use of immunosuppressive drugs, serious side effects of these medications dramatically increase the risk component of the ratio. However, this problem is overcome by the use of cells of autologous origin.

The feasibility of cell therapy includes the possibility of manufacturing the product in compliance with cell and tissue banking regulations and current good manufacturing practice principles, which requires complete control and traceability of all processes from the procurement of the raw materials to the delivery of the grafts, the reproducibility of graft manufacturing, the characteristics of the product, and strong quality assurance and quality control services.

In addition, the harvesting procedure should be simple and as non-invasive as possible. Feasibility of the implantation of the cell therapy product must be demonstrated in terms of easy handling (by the surgeon) and implantability in a dedicated implantation site.

Therefore, the aim of this study was to evaluate the safety and feasibility of two cell therapy products derived from ASCs in the context of hospital exemption.

## Materials and Methods

### Design of the study

Two autologous cell therapy products were studied: (1) a scaffold-free osteogenic 3D graft for the treatment of bone non-union (congenital pseudarthrosis/intercalary bone allograft implantation after tumor resection) and (2) a biological dressing for dermal reconstruction of non-healing chronic wounds (radionecrosis, drepanocytosis, and vasculitis).

**Fig 1:**
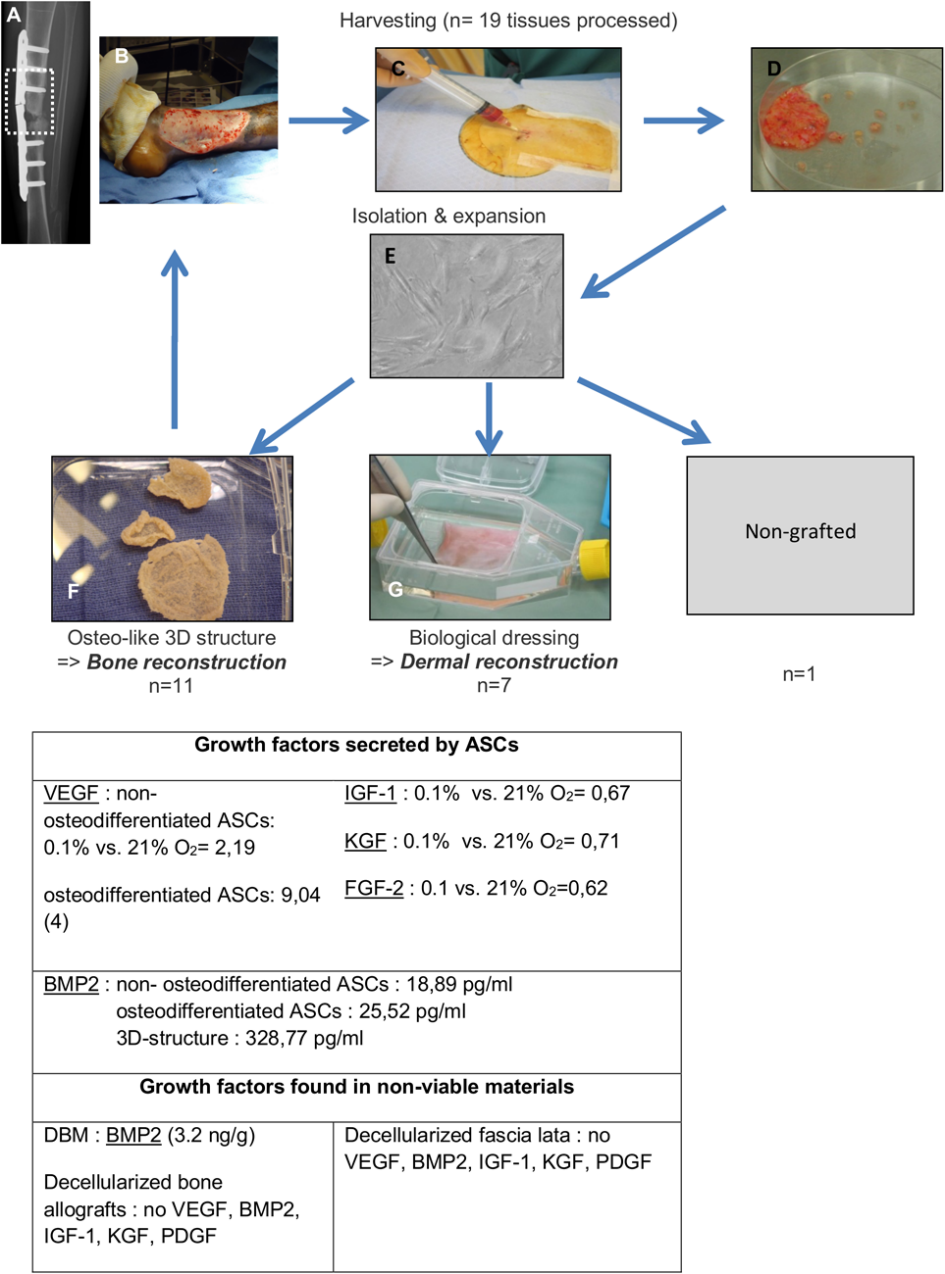
Autologous grafts manufactured. The schema of autologous ASC-derived cell therapy includes adipose tissue harvesting from a patient under local anesthesia. A: Bone non-union following allograft. B: Chronic wound on drepanocytosis. C: fatty tissue harvesting. D: ASC isolation. E: Expansion in culture plates. F: 3D osteogenic-like structure. G: Biological dressing. The table indicates the growth factors secreted by human ASCs. Results shown are the ratio of growth factors expressed in hypoxia (0.1% O_2_) versus normoxia (21% O_2_). VEGF = vascular endothelial growth factor; HGF = hepatocyte growth factor; PDGF = platelet derived growth factor; FGFb = fibroblast growth factor b.

These two cell therapy products were manufactured from autologous ASCs. Fatty tissue was harvested from the patient by a simple and rapid procedure under local anesthesia (lipoaspirate) or during a surgical procedure (pieces of fatty tissue were taken during tumor biopsy for diagnosis) and sent to the clean room for treatment. ASCs were then isolated, expanded *in vitro*, and, if necessary, differentiated and/or seeded on a scaffold. Once the culture process was completed, the cellular product was re-implanted in the patient (Fig 1).

This study was performed according to the Belgian Ministry of Health (AFMPS) guidelines for hospital exemption (and obtained the authorization by the national central authorities as the clinical number studies: ATMP-HE004 and ATMP-HE005). All procedures for tissue procurement and clinical studies (for adult and children patients) were approved by the Ethical Committee of the Medical Faculty (Université Catholique de Louvain) as the national authorization number: B40320108280 and B40320108542. All patients (adults and parents of the children) signed the consent to participate in the study after verbal and written information were received from the principal investigator of the study. All consent forms were included and archived in the Case Report Form for each patient (and for a duration of 30 years).

All materials were obtained from Lonza (Verviers, Belgium), Sigma-Aldrich (St. Louis, MO, USA), or Invitrogen (Carlsbad, CA, USA) unless otherwise noted.

### Clinical data of the patients

A total of 17 patients were included in the study (Fig 1) and 19 grafts were manufactured.

Eleven patients received a tridimensional stem cell autograft during orthopedic surgery. One additional patient with enchondromatosis did not undergo implantation because she presented with spontaneous consolidation. The orthopedic cases included six oncology patients (five males and one female): one patient with chondrosarcoma (OLS010) who received an allograft after tumor resection more than 2 years before being included in the study and five patients with bone tumors (three osteosarcomas: OLS001, OLS004, OLS005) and two Ewing sarcomas (OLS003 and OLS006 characterized by several clonal cytogenetic alterations) who underwent preoperative chemotherapy for a mean duration of 2 and 12 months before tumor resection. In these cases, anatomical reconstitution was performed with a metallic prosthesis and a human osteochondral allograft, respectively (Table 1). In addition, after failure of conventional therapies such as surgery (e.g., curettage, elongation, Fassier-Duval Telescopic system, intramedullary fixation, and Ilizarov fixation), iliac crest autograft, and demineralized bone matrix alone, five patients with non-union of bone due to congenital pseudarthrosis (n = 4; OLS002, OLS007, OLS009, OLS011) or acquired pseudarthrosis (in the context of erythroblastopenia, n = 1; OLS008) were given the option of receiving the osteogenic-like 3D structure (Table 1). One patient with congenital pseudarthrosis underwent grafting twice (OLS007 and OLS011).

**Table 1:** Clinical/manufacturing data associated with the implantation of the manufactured 3D osteogenic-like autologous grafts.

Patient ID	Clinical Indication	Clinical History and Previous Intervention	Age (yr)	Gender	Harvesting Site	Quantity of Fatty Tissue Harvested	Time to Obtain the Graft (d)
OLS001	Non-metastatic osteosarcoma grade III, left distal femur	Chemotherapy	11	M	Per-op	2.1 g	81
OLS002	Right tibio-fibular congenital pseudarthrosis without neurofibromatosis	Two nailings and one Ilizarov compression	11	F	Per-op	1.9 g	119
OLS003	Ewing's sarcoma, left diaphyseal femur	Bone non-union after allograft, reconstruction after tumor resection (1.5 yr previously)	9	M	Per-op	9.68 g	133
OLS004	Diaphyseal osteosarcoma grade III, left femur	3 sessions of thermocoagulation, curettage, trepanation, and chemotherapy	47	F	Peri-umb	16.8 g	48
OLS005	Osteosarcoma, left proximal tibia	Chemotherapy, 14-cm resection, and osteochondral allograft reconstruction	11	M	Per-op	0.3 g	80
OLS006	Ewing sarcoma, right proximal tibia	Chemotherapy	12	M	Peri-umb	1.1 g	143
OLS008	Iatrogenic non-union + eryblastopenia (Blackfan Diamond disease)	1 yr before ASCs isolation: tibial elongation and osteotomy	13	M	Per-op	7 g	111
OLS009	Congenital pseudarthrosis (type I neurofibromatosis), left ulna (+ radial shortening)	1.5 yr before ASCs isolation: pseudarthrosis resection, iliac crest graft, ulna elongation	6	F	Peri-umb	1 g	117
OLS010	Isolated chondrosarcoma grade I-II backdrop, left acetabulum to pelvic bone-ischiopubic branch	Chondrosarcoma resection and allograft (2.5 yr previously)	63	M	Peri-umb	9.8 g	108
OLS011	Congenital atrophic non-union (type IV Crawford classification), right tibia and fibula	2 yr before ASCs isolation: synostose resection, recurrent osteotomies, and iliac graft	9	M	Per-op	2.5 g	109
OLS007	Congenital atrophic non-union (type IV Crawford classification), right tibia and fibula	1.5 yr before ASCs isolation: fracture after fall	6	M	Per-op	0.6 g	130

Per-op: per operation. Peri-umb: peri-umbilical.

Seven cases of non-healing chronic wounds (>8 months of bad evolution) untreatable by classic therapies such as hyperbaric oxygen therapy, skin grafting, and active dressings (evolution of 13 to 46 months) were treated with a biological dressing made of autologous ASCs. One male patient with a chronic wound attributable to radionecrosis after irradiation for hystiosarcoma underwent grafting twice with the biological dressing (SBD001, SBD002). A dressing was also grafted to one male patient and to one female patient with non-healing ulcerations on drepanocytosis (SBD003) and systemic lupus erythemotosus (SBD004), respectively. One male patient presented with radionecrotic chronic wounds after irradiation for liposarcoma (SBD005) and another male patient had chronic ulcers after a full-thickness burn (SBD006). The lower limb wounds of this last case were covered by pedicled flaps, but a chronic wound developed at the edge of the area (evolution over 3 years). Finally, one male type 2 diabetic patient with multiple complications (retinopathy, microalbuminuria, macroangiopathy, neuropathy) was treated for his chronic wounds with the biological dressing (SBD007) (Table 2).

**Table 2:** Clinical/manufacturing data associated with the implantation of the manufactured biological dressings.

Patient ID	Clinical Indication	Clinical History and Previous Intervention	Age (yr)	Gender	Time Course (mo)	Quantity of Fatty Tissue Harvested (g)	Total Time of Culture (d)	Surface of the Wound Treated (cm^2^)
SBD001	Chronic wound, radionecrosis	Radionecrosis after adjuvant radiotherapy after sarcoma resection	46	M	8	59	83	12
SBD002	Chronic wound, radionecrosis	Radionecrosis after adjuvant radiotherapy after sarcoma resection	47	M	8	21.9	86	12
SBD003	Chronic wound, drepanocytosis	Homozygote drepanocytosis, 4 large supra malleolar wounds, no success with skin autograft	21	M	46	7.8	175	>250
SBD004	Chronic wounds, vasculitis (lupus erythematosus disseminated)	Systemic lupus erythematosus complicated by 4 large supra malleolar wounds, no success with skin autograft	33	F	27	20.8	131	>200
SBD005	Chronic wound, radionecrosis	Liposarcoma 44 yr ago with adjuvant radiotherapy, distal arteriopathy, chronic ulcer treated, no success with skin autograft	67	M	32	4.2	64	24
SBD006	Chronic wounds, full-thickness burn	For 46 yr, chronic ulcers in distal lower limbs after full-thickness burn, early coverage by pedicled flap after failure of primary skin autografts, no success with skin autograft	72	M	>36	8.6	127	~26
SBD007	Chronic wounds, diabetes (type 2)	Type 2 diabetes for 13 yr with multiple complications, 10 hospitalizations in the past 7 yr for soft tissue infections (secondary to leg and diabetic foot ulcers), no success with skin autograft	71	M	>240	8.6	35	200

Adipose tissue was harvested in the peri-umbilical zone for each patient.

### Graft manufacturing

#### Fatty tissue procurement and ASC isolation and expansion

Grafts were manufactured in the Endocrine Cell Therapy Unit (Center of Tissue and Cell Therapy, Cliniques Universitaires Saint-Luc, Brussels, Belgium), which is recognized by the Belgian Federal Agency for Medicines and Health Products as a clinical laboratory for processing ASC isolation. All ASC isolation and expansion procedures were performed in grade A air-laminated flow located in a grade B clean room (validated annually by ICCE SA, Elsene, Belgium, in accordance with the Belgium Ministry of Health recommendations and European directives, regulation no. 1394/2007 for advanced cell therapy products). The environment for cellular culture was controlled by weekly particle counting (in static and dynamic conditions; Lasair II Particle Counter; Particle Measuring Systems Germany GmbH, Darmstadt, Germany) and microbiological testing at each manipulation, as recorded in the graft report.

A mean of 9.7 ± 13.7 g of fatty tissue was harvested from each patient after submitting informed consent and serologic screening (anti-HIV-1, anti-HIV-2, anti-HCv, anti-HBc, anti-HBs, Ag HBs, CMV Ig, syphilis) and nucleic acid testing (HIV-1, HCV, HBV) by a simple subcutaneous biopsy or by lipoaspiration using the Coleman technique [30]. Adipose tissue was kept for a maximum of 90 min at 2°C to 8°C until tissue digestion and ASC isolation in line with the Belgian Federal Agency for Medicines and Health Products recommendations and the ISO 9001–2008 quality management system. Fatty tissue was digested with GMP collagenase ≥8 PZ U/ml (Serva Electrophoresis GmbH, Heidelberg, Germany) reconstituted in Hank’s balanced salt solution (with calcium and magnesium ions) at 37°C for 60 min. After digestion, the collagenase was inactivated in proliferation medium made of DMEM supplemented with 10% heat-inactivated fetal bovine serum (FBS), L-glutamine (2 mM), and antibiotics (penicillin 100 U/ml and streptomycin 100 μg/ml). Collected tissue was centrifuged for 10 min at 450×g. After filtration through a 500-μm mesh screen (Haver, Battice, Belgium), the tissue was centrifuged for 10 min at 450×g and then resuspended in proliferation medium. This initial passage of the primary cells was referred to as passage 0. After 24 to 48 h of incubation at 37°C at 5% CO_2_, cell cultures were washed with phosphate-buffered saline (PBS) and maintained, after sequential trypsinizations, in proliferation media up to passage 4. The selection of ASCs was performed based on their capacity to adhere to plastic culture flasks. Quality controls confirmed the mesenchymal phenotype and the purity of ASC preparations (see “Graft properties” section below).

### Specific cellular therapy product manufacturing and implantation

#### Osteogenic-like 3D structure

At passage 4, ASCs were incubated (in 150 cm^2^ culture flask) in osteogenic medium composed of the proliferation medium supplemented with dexamethasone (1 μM), sodium ascorbate (50 μg/ml), and sodium dihydrophosphate (36 mg/ml). After 15 to 18 days of ASC incubation, demineralized bone matrix (DBM) was added (10 mg/ml) to create the tridimensional structure. Human DBM (cortical bone particles, 200–700 μm, treated with HCl 0.6 N for 3 h and sterilized with 25 kGy by gamma irradiation; provided by the University Tissue Bank, University Clinical Hospital Saint-Luc, Brussels, Belgium), which is known for its osteoinductive properties, was added (10 mg/ml) to create the tridimensional structure.

The tridimensional graft was rinsed three times with transplantation medium (CMRL; Mediatec Inc., Manassas, VA, USA) without phenol red and without antibiotics or sera. The graft was finally placed in a sterile culture flask enclosed in three sterile plastic bags. The graft was then transferred at room temperature, for less than 15 min, to the operating room for implantation (Fig 1).

In case of tumor resection, the 3D graft was placed directly at the junction between the native host bone and the bone allograft or the growing prosthesis. In case of bone non-union, the tridimensional osteogenic graft was modeled to the ideal size of the bone defect and placed directly into the hole without any fixation material.

#### Biological dressing

At confluency at passage 3 or passage 4 (depending on the size of the wound), cells in proliferation medium were trypsinized, resuspended in 400 μl DMEM, and seeded on a human acellular collagen matrix (HACM) [31–33]. Fascia lata from selected donors were procured according to European/Belgian legislation regarding human body materials after human tissue donor screening based on clinical history, serological tests, and microbiological testing. The human fascia lata tendon was prepared as already described by using a process developed by the Tissue Bank of Cliniques Universitaires Saint Luc (Brussels, Belgium) [31] to obtain HACM containing no chemical residues (acetone/H_2_O_2_), with pH of the water last used for rinsing being between 7.00 and 7.84 and having less than 10% residual moisture, and sterilized by gamma irradiation at 25,000 Gy (Sterigenics, Fleurus, Belgium). HACM and ASCs were maintained in culture in 150 cm^2^ flasks until the collagen matrix was covered by ASCs (classically 3–4 weeks) [34]. On the day of implantation, the composite graft was rinsed three times with transplantation media (CMRL; Mediatec, Manassas, VA, USA).The dressing was finally placed in a sterile culture flask embedded in three sterile plastic bags with ASCs loaded on the upper side of the HACM. The graft was then transferred at room temperature to the operating room for implantation (Fig 1). In seven patients, wound debridement was performed by hydrosurgery (Versajet®; Smith & Nephew, Zaventem, Belgium) before the implantation to ensure a minimally contaminated wound bed. The composite graft was cut to an ideal size and oriented with the cell layer directly in contact with the wound surface. The HACM was then fixed with non-absorbable sutures. The composite graft integration was clinically assessed twice weekly. Vaselinated dressings were applied and changed daily.

### Safety

#### Microbiological safety

During the whole process of graft manufacturing, environmental and microbiological controls were performed. Environmental controls were performed during each manipulation of the cells by air sedimentation plates and total particle count. In addition, surface controls with contact plates in the clean room were performed weekly as well as before and after steps of ASC isolation and graft packaging. Microbiological testing was repeatedly performed at each media change (twice per week during the entire manufacturing of the graft) for aerobia, anaerobia, moisture, and yeast by BACTEC assays according to European Pharmacopeia 2.6.1. In addition, the sterility of the transport medium was checked after implantation in the operating room. Finally, endotoxin and mycoplasma assays were performed according to European Pharmacopeia 2.6.14 by Texcell SA (Evry, France) on the last cellular samples collected before graft delivery.

Microbiological testing was repeatedly performed at each media change (twice per week during the entire manufacturing of the graft) for aerobia, anaerobia, moisture, and yeast by BACTEC assays. In addition, total particle count was recorded during all manipulations.

### Genetic stability

Cytogenetic stability was studied by conventional karyotyping and fluorescent in situ hybridization (FISH) analyses to assess the oncogenic safety of cellular therapy products. Metaphase chromosomes were obtained according to standard protocols from cells at passage 1 and passage 4 and osteodifferentiated cells [9]. When possible, 20 reverse Trypsin-Wright G-banded metaphases were analyzed and karyotypes were reported according to the 2013 International System for Human Cytogenetics Nomenclature (ISCN 2013). Clonal aberrations were defined as at least two mitoses with the same chromosomal gain or structural anomaly and at least three mitoses with the same chromosomal loss. Fluorescent in situ hybridization was performed according to standard protocols [35] to detect aneusomies and aneuploidies (systematically researched by FISH for at least two loci) and the initial tumor-associated genomic abnormalities. In total, 17 different loci were analyzed on 11 different chromosomes with a classical commercial probe and two homemade BAC probes (S1 Table: Probes used for FISH analysis).

The thresholds were calculated following inverse beta law with a confidence interval of 99.9%.

### Adverse events

Safety was studied in terms of adverse events (local or systemic) with clinical (inflammation, wound infection) and biological (C-reactive protein, fibrinogen, white blood cell count) assessments (at day 0 and day 3). The long-term safety of the 3D osteogenic structure was investigated by imaging (X-ray or magnetic resonance imaging) between 1 and 54 months post-transplantation to assess any secondary ectopic malignant tissue development. Post-operative clinical examinations allowed evaluation of the long-term safety of the biological dressing. A biopsy of the zone treated with the biological dressing was also performed between 4 and 8 weeks post-implantation for histological analysis (neovascularization [Masson’s trichrome], inflammation [CD3/CD68 immunostainings], and fibrosis).

### Feasibility

The feasibility study was based on the ability to reproducibly obtain the desired graft (i.e., a 3D osteogenic-like structure from autologous ASCs and allogenic DBM or a biological dressing made of autologous ASCs and allogenic collagen matrix) in a sufficient amount to treat the lesion. The time between adipose tissue procurement and graft implantation was also assessed. The capability of developing quality control tests able to confirm the mesenchymal origin of ASCs and the optimal manufacture of the graft was evaluated. Surgical handling of the graft and the easy implantation procedure were considered. Finally, the ability to manufacture the graft in conditions in accordance with the Belgian quality guidelines (Belgian Federal Agency of Drugs and Health Product) was evaluated.

### Graft properties

From the 13^th^ patient, at the end of the proliferation phase (at passage 4), the mesenchymal lineage of ASCs was confirmed by flow cytometry. Cells were stained with saturating amounts of monoclonal CD90, CD44, CD45, CD73, and CD105 antibodies (BD Pharmingen, San Diego, CA, USA) conjugated with phycoerythrin or fluorescein isothiocyanate. At least 10,000 events were analyzed by flow cytometry (FACScan; BD Biosciences, Erembodegem, Belgium) with CellquestPro software. Percentages of cells expressing markers were calculated with a threshold corresponding to 95% of control cells. To confirm their multipotency, ASCs underwent adipogenesis, osteogenesis, and chondrogenesis by differentiation in specific adipocyte, osteoblast, and chondroblast induction media, respectively, as already described [13,14]. Alzarin red, Alcian blue (AppliChem GmbH, Darmstadt, Germany), and oil red staining were used to confirm osteogenic, chondrogenic, and adipogenic phenotypes, respectively, after differentiation.

A biopsy specimen (minimum of 8 mm^3^) of the osteogenic-like 3D structure harvested the day of the implantation was fixed in 4% paraformaldehyde overnight. Serial sections (5 μm thick) were made for staining with osteocalcin (anti-osteocalcin OC4-30 monoclonal mouse antibody at a dilution of 1:100; ABCAM, Cambridge, UK), extracellular matrix (by Masson’s trichrome), and mineralization (by Von Kossa) on each sample for all types of differentiated and non-differentiated cells (negative control). Quantification of the cellular portion, the neosynthesized extracellular matrix, and the DBM fragments via the calculation of the ratio [(DBM:extracellular matrix)/cell] was performed to evaluate the quality of the graft (the ratio should be between -1 and +1 to ensure sufficient quality) [17].

For the biological dressing, the cellular covering of the HACM was controlled (by an independent pathologist) 1 week before transplantation by a biopsy specimen of the composite graft fixed in 4% paraformaldehyde overnight and stained by hematoxylin and eosin and DAPI (4ʹ,6ʹ-diamidino-2-phénylindol; fluorescent molecule binding to adenine and thymine bases of DNA).

### Surgical handling of the graft

The easy handling of the graft and the capacity of the cellular therapy product implantation were evaluated by the surgeon. The graft should be easily manipulated and placed at the implantation site by the surgeon. Tridimentional ASC autografts should be malleable to fill bone (manually or by the use of a trochar), and the size and shape of the biological dressing should be adaptable and suturable on the wound bed by the surgeon.

## Results

### Safety

#### Microbiological safety

**Fig 2:**
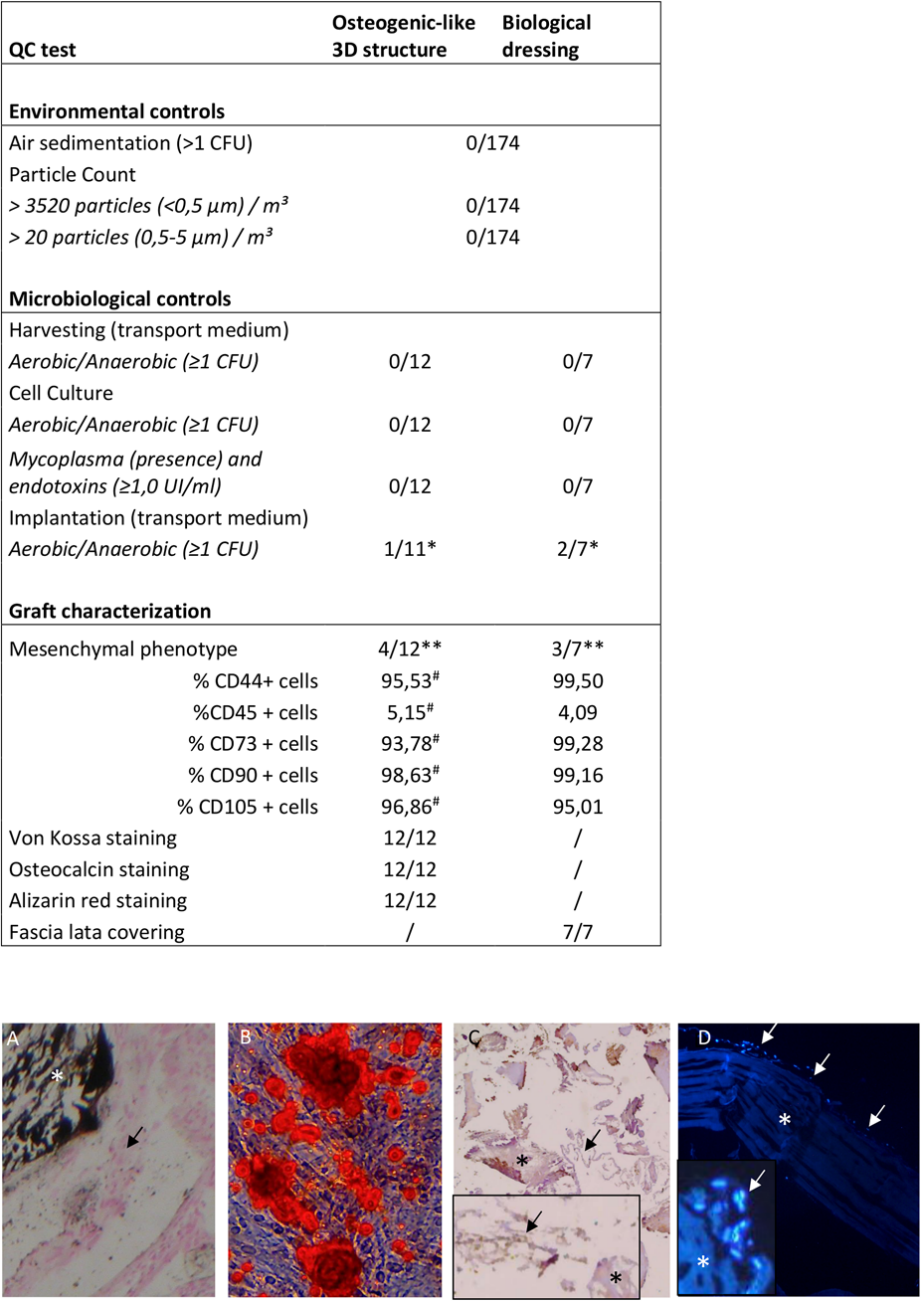
Quality controls of the manufactured grafts. Environmental controls including air sedimentation and total air particle count were performed during each manipulation of the product. Microbiological controls (sterility) were performed on harvesting transport medium, on cell culture medium at each cell medium change, and on the implantation transport medium. In addition, mycoplasma and endotoxins were measured on the last medium change before implantation. Fluorescence-activated cell sorting and histological assessments allowed characterization of the product. *Staphylococcus aureus, *Staphylococcus epidermidis*, and corynebacterium striatum contaminations were detected. However, the contamination occurred in the operating room and did not affect the product. Fluorescence-activated cell sorting analyses were not performed for bone reconstruction for the first eight patients or for dermal reconstruction for the first four patients. A: Von Kossa staining of the 3D osteogenic-like structure (*DBM particle; arrows: ASCs + extracellular matrix). B: Alizarin red (RA) coloration of osteodifferentiated ASCs. C: Osteocalcin staining of the 3D osteogenic-like structure (*DBM particle; arrows: ASCs + extracellular matrix). D: DAPI staining of ASCs seeded on the fascia lata (*fascia lata; arrows: ASCs on the scaffold).

Environmental controls, including laboratory surface controls, particle count, and air sedimentation, were performed during all steps of graft manufacturing. Three results (Fig 2) were not below the authorized limits. *Staphylococcus aureus*, *Staphylococcus epidermidis*, and corynebacterium contaminations of the transport medium were noted after implantation; however, the medium was sterile when it left the production unit, indicating contamination in the operating room.

### Genetic stability

Genetic studies (karyotype and/or FISH) were available for 15 different manufactured grafts (not performed in four grafts). First, of the six oncologic patients, the initial tumor-associated genomic anomalies were not recovered after cell proliferation and differentiation.

For nine grafts, genetic results were informative for stages of “end of proliferation” and “differentiation” (S2 Table: Genetic analysis results on manufactured grafts). No aberrations were detected at these stages in four grafts (OLS003, OLS005, OLS009, and OLS011). Genetic abnormalities induced during the proliferation phase disappeared or were reduced after differentiation in two cases (OLS002 and OLS010). For OLS010, a stable balanced reciprocal translocation was detected in 52% of cells, as was trisomy 7 in 7% of cells at the end of the proliferation phase. At the end of the differentiation phase, the translocation was reduced to 31% of cells and trisomy 7 disappeared.

Two different translocations involving chromosome 15 (8% of cells) and trisomy 7 in 20% of cells were detected in proliferation and differentiation phases, respectively, in patient OLS006 (S2 Table). However, cells were maintained in the proliferation phase for 3 additional months without any abnormalities detected. In addition, the initial anomaly disappeared after culture. A very minor deletion of the short of chromosome 3 was detected in the differentiation phase of OLS008 (2 out of 40 analyzed cells, which is theoretically under the threshold for the definition of a chromosomal clonal aberration).

#### Peri-/post-operative security (short- and long-term)

No notable adverse events were noted during and after the implantation procedure. Three cases of infection were noted. However, these infections affected the skin or the synthetic material, but the cellular product was not implicated (Tables 3 and 4).

**Table 3:** Complication data associated with the implantation of the manufactured biological dressings.

Patient ID	Maximum Follow-up (mo)	Short-Term Complications	Long-Term Complications
OLS001	54	Delayed wound healing (at 11 mo, scar OK), no infection	No prolonged inflammation, no infection, no neoplastic development
OLS002	2	Optimal wound healing, no infection	No prolonged inflammation, no infection, no neoplastic development
OLS003	1	Optimal wound healing, no infection	No prolonged inflammation, no infection, no neoplastic development
OLS004	27	1 mo: Lateral external surface, left tight oozing (abscess femoral scar)	Subcutaneous collection (*Staphylococcus aureus*) easily treated, scar OK at 8 wk, no infection, no neoplastic development
OLS005	48	2 mo: Small skin necrosis on the internal scar, evolution toward a fibrin crust, wide debridement + care until complete healing, no infection	No prolonged inflammation; 10 mo: resection following cellulite on the outer side of the leg (without collection); no neoplastic development
OLS006	48	6 wk: Optimal wound healing, no infection	No prolonged inflammation, no infection, no neoplastic development
OLS007	37	Follow-up 2 wk to 3 yr: beautiful scar, no infection	No prolonged inflammation; 9 mo: redness of leg on old port pin, free flowing, clean, crust formed, CRP <0.1, normal white blood cells; no obvious signs of infection, no neoplastic development
OLS011	13	4 mo: Optimal wound healing, no infection	No prolonged inflammation; 4 mo: plate and nail fracture, reconstruction by autologous vascularized fibula at 6 mo; 6 mo: perioperative *S*. *epidermidis* detection, no neoplastic development
OLS008	29	Not determined	No prolonged inflammation, no infection, no neoplastic development
OLS009	10	Optimal wound healing, no infection	No prolonged inflammation, no infection; 6 mo: redness; 7 mo: decrease in external inflammatory signs; 10 mo: slightly elevated CRP and white blood cells to 14,000; 10.5 mo: swelling next to the plate; 11 mo: *S*. *aureus* sensitive to oxacillin
OLS010	47	Optimal wound healing, no infection	No prolonged inflammation, no infection, no neoplastic development

**Table 4:** Complication data associated with the implantation of the manufactured 3D osteogenic-like graft.

Patient ID	Maximum Follow-up (mo)	Short-Term Complications	Long-Term Complications
SBD001	22	Complete healing maintained up to now (>22 mo), no infection	No prolonged inflammation, no infection, no neoplastic development
SBD002	22	Complete healing maintained up to now (>22 mo), no infection	No prolonged inflammation, no infection, no neoplastic development
SBD003	35	Healing for >6 mo (recurrence or poor control of the systemic disease), no infection	No prolonged inflammation, no infection, no neoplastic development
SBD004	38	Healing for 2 mo (recurrent/systemic disease), no infection	No prolonged inflammation, no infection, no neoplastic development
SBD005	9	Complete healing, no infection	No prolonged inflammation, no infection, no neoplastic development
SBD006	7	Partial healing (half of the treated area), no infection	No prolonged inflammation, no infection, no neoplastic development
SBD007	6	No notable wound healing, no complications	No prolonged inflammation, no infection, no neoplastic development

One patient with osteosarcoma showed recurrence of her tumor. However, anatomopathological reports showed that tumoral cells were present at the resection margins, indicating an insufficient initial tumor resection. In addition, because the implanted cells lost their oncogenic phenotype after differentiation, the implanted cells are seemingly not responsible for this recurrence.

**Fig 3:**
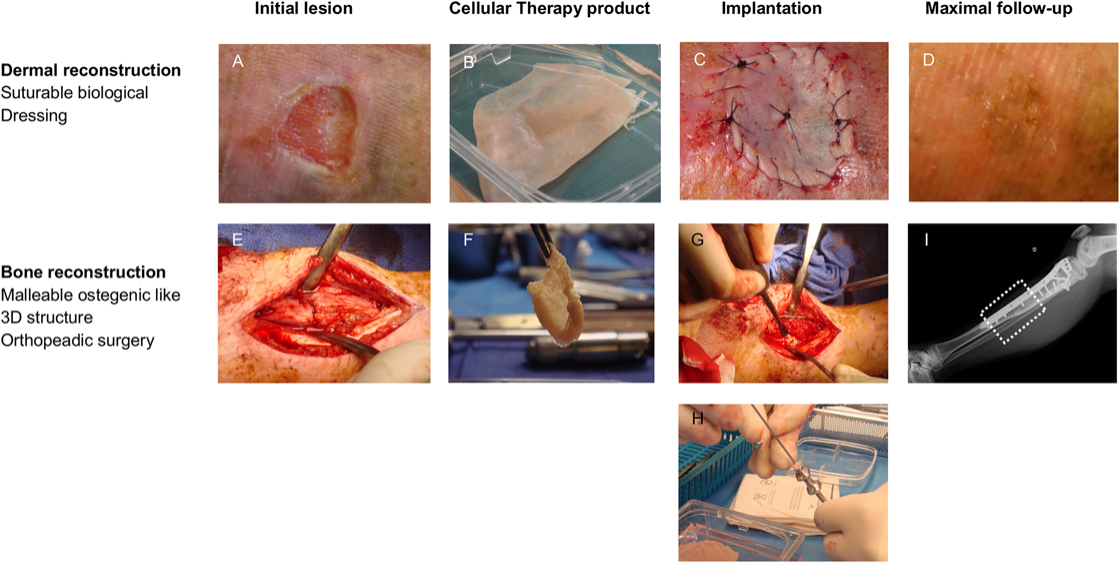
Pre-, peri-, and post-operative pictures of the treated cases. A: Chronic wound (12 cm^2^) on radionecrosis (SBD001). B: Autologous biological dressing before implantation. C: Implantation of the biological dressing (SBD001); note that this was easily suturable. D: Follow-up at 5 months after implantation (SBD002) (follow-up at 22 months was similar) (9). E: Bone defect on congenital pseudarthrosis (OLS002). F: Autologous osteogenic-like 3D structure before implantation. Implantation of the osteogenic-like 3D structure (G) manually (OLS002) or (H) through a trocar (OLS003). I: Follow-up at 46 months after implantation (OLS006).

In contrast, to date, for the 17 other grafts for orthopedic surgery and for the biological dressing, no formations of ectopic tissues or neoformation of tumors were observed for a maximum of 54 months and 38 months, respectively (Fig 3).

### Feasibility

In all patients, a mean of 9.7 g (range, 0.3–59 g) of adipose tissue was easily harvested without any notable complication. When culture conditions were optimal (no excess of blood during AT procurement and 5% CO_2_ in the cell culture incubator), ASC isolation, proliferation, and manufacturing of the three grafts types were reproducibly performed in 100% of cases.

A mean of 107 ± 28 days was needed to obtain a tridimensional osteogenic graft (Table 1), whereas the biological dressing was obtained after a mean of 100 ± 47 days (Tables 1 and 2). In 100% of cases, the timing imposed by the surgeon was adequate.

Grafts were manufactured in a sufficient amount to fill the defects (for osteogenic-like structures) or cover the entire wound surface, even for large wounds (>200 cm^2^ for biological dressings).

ASCs were confirmed as stem cells by a positive shift in CD90, CD44, CD73, and CD105 and by no shift in CD45 expression by flow cytometry. ASCs presented a fibroblast phenotype in the proliferation medium. The multipotency of ASCs was confirmed by their differentiation in adipocytes, osteocytes, and chondrocytes.

All 12 osteogenic-like 3D structures presented osteocalcin expression, synthesis of extracellular matrix, and mineralization (by Von Kossa) on each sample for all types of differentiated and non-differentiated cells (negative control). The histomorphological score (DBM—extracellular matrix)/number of cells) was always between -1 and 1, as required [17].

The hematoxylin and eosin and DAPI staining confirmed the cellular covering of the HACM.

The Belgian quality guidelines (Belgian Federal Agency of Drugs and Health Product and ISO9001) were followed during the processing of the whole graft from the procurement of the adipose tissue (informed consent, viral screening of the donor, and absence of microorganisms in the harvested tissue) to the delivery of the graft (conformity of quality controls, timing of manufacturing).

The osteogenic-like 3D grafts formed prehensile structures easily malleable by the surgeon to fill bone defects, manually or through the use of a trocar. However, these grafts were stable enough to avoid any dissemination outside their implantation sites (Fig 3).

The biological dressings were able to be manipulated and their shapes were adaptable by the surgeon. The dressings were resistant and suturable on the wound beds (Fig 3).

## Discussion

This study demonstrates (i) the safety of cell therapy comprising differentiated and undifferentiated ASCs, (ii) the capacity to reproduce two distinct cell therapy products available for two different surgical applications, and (iii) the importance of the pathophysiology of the disease in the context of cell therapy.

The first question addressed in this study was to ensure the safety of the procedure, even in an oncologic field. No notable undesirable effects were observed in grafted patients after ASC collection or during short-term or long-term periods after the implantation of the product. However, there are challenges concerning the genetic safety of cell therapy. Although malignant transformation of MSCs has not been noted to date in clinical trials using culture-expanded autologous and allogenic human MSCs [36–40], controversy exists concerning the potential of spontaneous transformation of MSC after prolonged *ex vivo* culture. *In vitro* studies have shown that MSC expansion may be associated with chromosomal alterations [41] and genetic instability and lead to tumor development in treated animals [42]. However, it was demonstrated that ASCs, up to passage 16, do not present clonal transformation [16], whereas in the present study cells were not used for more than five passages. Aneusomies such as trisomy 7 were detected in several cases. It is known to be induced in proliferative cultured human MSCs and to have no obvious impact on the safety of the cells, as previously reported by Tarte et al. [43]. The cells from this patient were maintained in culture for 3 additional months without any anomalies detected. Minor tetraploidy and translocations were also noted in the proliferation phase in one and two patients, respectively, but it was reduced or disappeared after differentiation. Aneuploidy, such as tetraploidy, is known to occur in cultured MSCs without any oncogenic significance [36]. Genetic analyses using both techniques (the pangenomic karyotype and targeted FISH on at least two different loci) did not show the major expansion of a clone at the differentiation phase, although some aberration appeared at the proliferation phase. A hypothesis explaining the loss of abnormal genotype after differentiation could be that proliferative cells with genetic alteration lose their capability to differentiate, leading to a natural positive selection of normal cells. Moreover, the low resolution of the karyotype does not preclude the absence of induced oncogenic mutations through the process of culture. Because validation of the use of a gene panel has not been already accomplished by NGS, the karyotype remains the reference technique for pan-genomic analysis in search of minor clones coupled with FISH. Imaging procedures and clinical examinations did not detect any tumor formation due to cell transplantation up to 4 years post-implantation.

The second concern of this study was the feasibility of the procedure, including the adequacy for clinical demand, the reproducibility of the procedure, and the compliance with current regulatory requirements. In addition to their intrinsic physiological properties, grafts should be easily implantable by the surgeon. Both types of grafts were easily handled by the surgeon for treating bone and skin lesions. The tridimensional ASC autograft was easily malleable to fill bone defects. It was prehensile with forceps and could be placed through a trocar. The implantation of ASCs for the treatment of chronic wounds was limited by the difficulty in maintaining cell contact with the wound. Therefore, ASCs were seeded on a collagen scaffold with a size and shape adaptable by the surgeon and suturable on the wound bed. The collagen support is resorbable in approximately 6 weeks, thus preparing the wound for a prospective skin graft. Improvements of the biological dressing need to be made to simultaneously promote both dermal and epidermal reconstruction. The grafts were reproducibly manufactured in a defined time. However, the latest is relatively long and should be reduced by improving culture conditions.

The cellular therapy products manufactured should be clinical grade. In this study, although the current good manufacturing practice was not fully achieved, Belgian national recommendations were followed. The human and raw materials were traced; all manipulations were thoroughly documented and regular quality controls were performed for the products and their environment. These controls allowed confirmation of the sterility of the graft and the detection of several dysfunctions and graft exclusion before implantation to guarantee patient safety. Ten grafts were rejected because the quality controls were not optimal. After analysis, it appeared that too much blood in the fatty tissue harvested inhibited ASC proliferation and led to the selection of another cell phenotype. In addition, poor regulation of the levels of carbon dioxide in the incubator prevented the formation of the 3D structure (pH higher than 7.6) (data not shown).

Finally, the adequacy between the expected theoretical effects of the graft, based on growth factors secreted by ASCs (VEGF, BMP2, FGF-2, KGF, IGF-1), and the observed clinical efficacy need to be verified. It appeared that ASC-based cell therapy products demonstrated optimal efficacy in clinical indications, where they were used to treat a local lesion in an unfavorable environment (lack of tissue vascularization, poor cellular recolonization, growth factors depletion, inflammation), but in the absence of a systemic physiopathology. The 3D osteogenic-like structure allowed bone consolidation for up to 4 years without any notable complications in oncologic patients with tumor resection (data not published). These non-consolidations after tumor resection are local lesions not associated with systemic pathologies or unfavorable condition (except the chemotherapeutic treatment). In contrast, more variable results were noted in patients with systemic diseases, such as congenital pseudarthrosis, characterized by an absence of a local favorable environment (low level of local and systemic growth factors, such as fibroblast growth factor 2) [44] or Blackfan-Diamond syndrome (erythroplastopeny). Similarly, the use of a biological dressing to treat chronic wounds allowed complete healing of wounds after radionecrosis or burn, which are local lesions, although it was not indicated in the case of systemic blood/vascular diseases (drepanocytosis, vasculitis, diabetes) [16]. The specific indications of each cell product should therefore be investigated, probably by selecting local lesions not associated with systemic pathologies.

In conclusion, we have demonstrated that cell therapy products obtained from autologous ASCs are feasible and safe for several reasons. The grafts could be reproduced in the desired form, in a defined timeframe, and in a sufficient amount to treat the lesion. Quality control tests were able to confirm the mesenchymal origin of ASCs, the optimal manufacture of the graft, the genetic stability of the cells, and the microbiological safety of the product. Clinical grade production guidelines were followed. The possibility of harvesting adipose tissue for all patients as well as the easy surgical handling of the graft and the easy implantation procedures were confirmed. The procedures were not associated with serious side effects or adverse events.

## Supporting Information

S1 Table Probes used for FISH analysis. (DOC)

S2 Table Genetic analysis results on manufactured grafts. (DOC)
