# Supplementary material for: Human Adipose-Derived Mesenchymal Stem Cells in Cell Therapy: Safety and Feasibility in Different "Hospital Exemption" Clinical Applications
Source: PLoS One. 2015 Oct 20;10(10):e0139566. doi: 10.1371/journal.pone.0139566 (PMC4615620; doi:10.1371/journal.pone.0139566)
Supplement: S1 Table — (DOC) [file pone.0139566.s001.doc]

S1 Table : Probes used for FISH analysis

| Patient ID | Probes used for FISH analysis |
| --- | --- |
| OLS001 | V_1cen/D1Z5, V_7cen/D7Z1, V_8cen/D8Z2, V_9q34/ABL1, K_ON_12cen/D12Z3, K_ON_12q15/MDM2, V_13q14/RB1, V_14q32/IGH@, V_18q21/BCL2, V_17cen/D17Z1, V_17p13/TP53, V_22q11/BCR et V_22q12/EWSR1 |
| OLS002 | V_21q22/LSI-21, V_7cen/D7Z1 |
| OLS003 | V_7cen/D7Z1, V_8cen/D8Z2, V_22q12/EWSR1 |
| OLS004 | V_7cen/D7Z1, V_8cen/D8Z2, V_13q14/RB1, V_17p13/TP53, V_9p21/CDKN2A/CDKN2B, V_9cen/D9S1752, C_1p36.33/T1p + V_1q44/T1q |
| OLS005 | V_7cen/D7Z1, V_CEP 8, V_RB1, V_17p13/TP53, V_9p21/CDKN2A/CDKN2B, V_9cen/D9S1752, K_ON_12cen/D12Z3, K_ON_12q15/MDM2 |
| OLS006 | C_ 11q24/ FLI1-22q12/ EWSR1, V_7cen/D7Z1, V_8cen/D8Z2, V_ D12Z3/CEP12, RP11-111L13 & RP11-705L22 / FOXP1/3q13 |
| OLS007 & 11 | V_7cen/D7Z1, V_8cen/D8Z2, RP11-111L13 & RP11-705L22 / 3q13/FOXP1 |
| OLS008 | V_7cen/D7Z1, V_8cen/D8Z2, RP11-111L13 & RP11-705L22 / 3q13/FOXP1 |
| OLS009 | V_7cen/D7Z1, V_8cen/D8Z2, RP11-111L13 & RP11-705L22 / 3q13/FOXP1 |
| 0LS010 | K_ON_1p36/1cen, _7cen/D7Z1, V_8cen/D8Z2, V_TP53, V_CEP 17 |
| SBD005 | V_7cen/D7Z1, V_8cen/D8Z2 |
| SBD006 | V_7cen/D7Z1, V_8cen/D8Z2 |

Probes designed by « V- »  are from Abbot SA, Wavre, Belgium

Probes designed by « K- »  are from Kreatech Diagnostics, Amsterdam, The Netherlands

Probes designed by « C- » are from Cytocell, Cambridge, UK
