# Supplementary material for: Human Adipose-Derived Mesenchymal Stem Cells in Cell Therapy: Safety and Feasibility in Different "Hospital Exemption" Clinical Applications
Source: PLoS One. 2015 Oct 20;10(10):e0139566. doi: 10.1371/journal.pone.0139566 (PMC4615620; doi:10.1371/journal.pone.0139566)
Supplement: S2 Table — (DOC) [file pone.0139566.s002.doc]

**S2 Table : Genetic analysis results on manufactured grafts.**

| **ID patient** | **Early proliferation** | **End of proliferation** | **Differentiation** |
| --- | --- | --- | --- |
| OLS001 | Not done | Not done | K : 46,XY[20]  F : Normal for 13 tested loci on 10 different chromosomes  * |
| OLS002 | K : Failure | K : 47,XX,+21[1]/46,XX[8]  F : + 21 (7,5%) | K : 46,XX[20]  F : Normal for 2 tested loci on 2 different chromosomes (7 & 21) |
| OLS003 | Not done | K : Failure  F : Normal for 3 tested loci on 3 different chromosomes (7, 8, 21)  * | K : 46,XX[20]  F : Normal for 3 tested loci on 3 different chromosomes (7, 8, 21)  * |
| OLS004 | Not done | Not done | K : Failure  F : Normal for 8 tested loci on 5 different chromosomes  * |
| OLS005 | Normal  * | K : 46,XY[50]  *chrt(3)(q13)[11]  F : Normal for 8 tested loci on 5 different chromosomes  * | K : 46,XY[20]  F : Not done |
| OLS006 | Not done | K : 46,XY,t(14;15)(q32;q11.2)[6]/46,t(X;15)(p11.2;q22)[2]/46,XY[94]  F : + 7 (13%)  Normal for 2 other tested loci on 2 different chromosomes  * | K : 47,XY,+7[10]/46,XY[26]  F : + 7 (19%)  Normal for 7 other tested loci on 7 different chromosomes  * |
| OLS006 + 3 Months |  | K : 46,XY[22]  F : Normal for 2 tested loci on 2 different chromosomes (7, 8) | K : Failure  F : Normal for 2 tested loci on 2 different chromosomes (7, 8) |
| OLS007 | Not done | K : 46,XY,+7[2]/46,XY[30]  F : + 7 (6.5%)  Normal for 2 other tested loci on 2 different chromosomes | K : 46,XY[18]  F : + 7 (6.5%)  Normal for 2 other tested loci on 2 different chromosomes |
| OLS008 | Not done | K : 46,XY[40]  * del(3)(p14)[1]  F : Normal for 3 tested loci on 3 different chromosomes (3, 7, 8) | K : 46,XY,del(3)(q14)[2]/46,XY[38]  F : Normal for 2 tested loci on 2 different chromosomes (7, 8) |
| OLS009 | Not done | K : 46,XX[20]  F : Normal for 3 tested loci on 3 different chromosomes (3, 7, 8) | K : 46,XX[20]  F : Normal for 3 tested loci on 3 different chromosomes (3, 7, 8) |
| OLS010 | Not done | K : 46,XY,t(7;8)(p22;q22)[32]/46,XY[29]  F : + 7 (7%)  Normal for 5 other tested loci on 3 different chromosomes  * | K : 46,XY,t(7;8)(p22;q22)[5]/46,XY[11]  F : Normal for 6 tested loci on 4 different chromosomes  * |
| OLS011 | K : Failure  F : Normal for 2 tested loci on 2 different chromosomes (7, 8) | K : Failure  F : Normal for 2 tested loci on 2 different chromosomes (7, 8) | K : Failure  F : Normal for 2 tested loci on 2 different chromosomes (7, 8) |
| SBD001 | Not done | Not done | Not done |
| SBD002 | Not done | Not done | Not done |
| SBD003 | Not done | Not done | Not done |
| SBD004 | Not done | Not done | Not done |
| SBD005 | K : Failure  F : +7 & +8 (13%)* | K : 46,XY[6]  F : Failure | Not done |
| SBD006 | K & F : Failure | K : 46,XY[10]  F : Failure | Not done |
| SBD007 | K & F : Failure | K : Failure  F : +7 & +8 (13%)** | Not done |

K : Karyotype analysis

F : FISH analysis

* : No detection of the initial tumor associated genomic abnomalities

** : on a very small number of cells (23)
